# On the Corrosion Mechanism of CO_2_ Transport Pipeline Steel Caused by Condensate: Synergistic Effects of NO_2_ and SO_2_

**DOI:** 10.3390/ma12030364

**Published:** 2019-01-24

**Authors:** Le Quynh Hoa, Ralph Baessler, Dirk Bettge

**Affiliations:** Federal Institute for Materials Research and Testing (BAM), Unter den Eichen 87, 12205 Berlin, Germany; Ralph.Baessler@bam.de (R.B.); Dirk.Bettge@bam.de (D.B.)

**Keywords:** carbon capture, utilization, and storage (CCUS) technology, corrosion, condensate, electrochemical characterization, pitting corrosion, impurities, carbon steel

## Abstract

To study the effects of condensed acid liquid, hereafter referred to as condensate, on the CO_2_ transport pipeline steels, gas mixtures containing a varying concentration of H_2_O, O_2_, NO_2_, and SO_2_, were proposed and resulted in the condensate containing H_2_SO_4_ and HNO_3_ with the pH ranging from 0.5 to 2.5. By exposing the pipeline steel to the synthetic condensate with different concentration of acidic components, the corrosion kinetic is significantly changed. Reaction kinetic was studied using electrochemical methods coupled with water analysis and compared with surface analysis (scanning electron microscopy (SEM), energy-dispersive X-ray spectroscopy (EDS), and X-ray diffractometry (XRD)) of corroded coupons. The results showed that, although the condensation of NO_2_ in the form of HNO_3_ causes faster general corrosion rate, it is the condensation of SO_2_ in the form of H_2_SO_4_ or the combination of SO_2_ and NO_2_ that may cause much more severe problems in the form of localized and pitting corrosions. The resulting corrosion forms were depended on the chemical nature of acids and their concentration at the same investigated pH. The effects of changing CO_2_ flow rate and renewing condensate on pitting corrosion were further studied.

## 1. Introduction

Carbon dioxide has been identified as one of the main contributors to global warming, leading to a 20 cm increase of average sea level from 1901 to 2010. To minimize the possibility of severe climate changes, the European Union and G8 have set a target to reduce at least 80% of greenhouse gas (GHG) emissions by 2050. Carbon Capture, Utilization, and Storage (CCUS) has been proposed and proven as a technology of choice for the mitigation of CO_2_ emissions and to reach the GHG reduction target [1,2,3,4,5]. Many capture technologies are now available for industrial applications such as pre-, post-combustion capture, oxyfuel combustion and solvent absorption [6,7,8,9]. However, they are not always directly installed at the CO_2_ storage/reuse sites, indicating a need for CO_2_ transportation network [10]. As the reliability and cost-effectiveness of the pipeline transport network are crucial to the overall operability and resilience of the CCUS system, it is vital to realize the possible corrosion risks of the employed pipeline steels corresponding to the impurity level of the gas source. 

Recent studies have shown that even at a very low concentration of impurities (such as 30 ppm_v_ SO_2_, 32 ppm_v_ of NO_2_, 70 ppm_v_ O_2_ and 90 ppm_v_ H_2_O) the formation and condensation of sulfuric and nitric acids in dense phase CO_2_ are possible and observable [11,12,13,14]. Carbon steel, a primary commercial material for constructing CO_2_ transportation pipelines, has low corrosion resistance to carbonic acid and these condensed acids. Depending on the type of capture technology (pre-combustion, oxyfuel or post-combustion), the concentration of each impurity in the captured CO_2_ stream will vary. The corrosiveness is not, however, only dependent on the content of each captured CO_2_ stream, but also the interactions between impurities from these sources when they are mixed within the network pipeline system, leading to bulk phase reactions and form aqueous phases. Up to now, a clear regulation procedure, which defines the maximum acceptable level of impurities and the combination of them for each employed pipeline steels, is lacking. The most effective mitigation methods are either removing water sufficiently (down to 50 ppm_v_) to prevent aqueous phases and excessive corrosion rates under the presence of other impurities or using corrosion-resistant alloys (CRAs). Both however significantly increase the cost. Further risks are a malfunction of dehydration unit and depressurization process to temporally shut down the pipeline, where the temperature of the system decreases (Joule-Thomson effect), along with an increased concentration of many impurities (including water, SO_2_, and NO_2_) in the remaining liquid phase. Thus, there is a need to quantify the increased corrosivity after depressurization to ensure safe operations. 

In our previous study, a screening experiment was conducted by mixing pure CO_2_ gas with a varying concentration of water 600, 1000, and 8000 ppm, 1.8% O_2_ and other components (SO_2_, NO_2_) [15,16]. Each time, the mixture was then fed (1.5 L min^−1^) into a glass-covered reactor containing 12 coupon-shaped specimens for 120–600 h at 278 K (to simulate the subterranean pipeline transport). From these experiments, to study the effect of condensate on the transport pipeline steel, a “worst-case scenario” gas mixture, containing 2.5% H_2_O, 1.8% O_2_, 1000 ppm NO_2_, and 220 ppm SO_2_, was proposed and resulted in the condensate containing 0.114 M H_2_SO_4_ and 0.0184 M HNO_3_ (pH 2.13) [16]. The major result is that the water content strongly affects to the corrosion rate, and together with the gas impurities, forming a highly corrosive acidic condensate that leads to a dramatic increase of corrosion process, even with the pipeline made of high-alloyed steels [15,16,17]. 

Following that study, we now focus on the role of each gas impurity, when the condensate is formed, by investigating the role of each acidic component on the corrosion behaviors of the commercialized pipeline-steel. Furthermore, the varying combination of these acidic components was made and exposed to the testing material and subsequently both the corroded coupons and the liquid products were analyzed to elucidate the corrosion kinetic. Effects of CO_2_ stream flow rate and renewal of condensate were studied to reveal their impacts on corrosion behaviors of the pipeline steel. The mechanism of corrosion reaction and benchmark for the impurity was proposed based on the exposure and electrochemical experiments, which were again compared with the test in a gaseous reaction vessel. 

## 2. Materials and Methods 

Carbon steel L360NB was chosen since this is the commercialized and realistic material for CO_2_ transport pipeline. The chemical composition of this steel is shown in Table 1. From the as-received pipeline sections, testing coupons were machined to the size of 20 mm × 15 mm × 5 mm. Typical sample preparation included mechanical polishing using 60, 120, and finally, 320 grit silicon carbide abrasive papers, cleaning with ethanol and degreasing with acetone and drying using nitrogen gas prior to every test. The mass and the dimensions of the specimens were measured for weight loss calculation. Experimental condition is summarized in Table 2. 

All tests in this study were conducted in a system consisting of three double-walled glass cells connected to a cooling system (Alpha RA8, Lauda, Lauda-Königshofen, Germany) to maintain the temperature at 278 K (5 °C). Each cell has 4 ports to purge the CO_2_ gas (purity > 99.995%, Linde AG, Germany) (50–60 mL/min) and to insert working, counter, and Ag/AgCl reference electrodes. In the case of exposure test, the coupon was hanged on the Teflon wire, while in the electrochemical test, the coupons were weld to a stick made of high-alloyed material. Before each test, 500 mL condensate was used and purged strongly with Ar (purity > 99.998%, Linde) for 30 min, then 1 h with CO_2_ to reach the saturation state and stable pH (≈1.9). 

### 2.1. Exposure and Electrochemical Test Setup 

A new coupon was used for each electrochemical measurement. A standard three electrode system consists of the tested coupon as a working electrode, a Ti/TiO_2_ counter electrode, and a Ag/AgCl reference electrode. Electrochemical measurements were carried out using a Gamry potentiostat “Reference 600”. The electrochemical measurements include the measurements of free corrosion potentials (open circuit voltage) and at the same time impedance measurements every 24 h. The electrochemical impedance spectroscopy (EIS) measurements were carried out using 10 mV amplitude perturbations and a frequency range of 10 kHz to 10 mHz at open circuit potential. Cyclic potentiodynamic polarization was applied by external DC voltage. The polarization experiments were started within 1 h after immersion, a scan rate of 1 mV s^−1^ was chosen and data were acquired at a rate of 1 Hz. The polarization scans were started at 20 mV cathodic from open circuit potential (OCP), then passing the OCP in anodic direction until reaching a current density of 10 mA, which was chosen arbitrarily as an unacceptable current, and finally reversed back into cathodic direction until intersection with the forward branch. 

### 2.2. Analyses

The anions in condensate were analyzed with Ion Chromatography (IC) Metrohm 883 Basic IC plus with column Metrosep A Supp5, and the cations were analyzed with Atomic Absorption Spectroscopy (AAS) (ZEEnit 600, Analytik Jena AG, Jena, Germany). Optical microscopic images were recorded by a Axioplan 2 imaging microscope with Zeiss AxioCam HRc camera (Carl Zeiss, Jena, Germany), and higher magnifications were obtained with a scanning electron microscopy (VEGA3, TESCAN, Dortmund, Germany), equipped with backscatter electron detector (BSE), in-lens detector and energy dispersive X-ray spectroscopy (EDS). The corroded coupons were also examined by X-ray diffraction analysis (XRD; Rigaku Ultima IV with D/teX Ultra detector, Tokyo, Japan). The corrosion rate was calculated by weight-loss measurement according to the standard ASTM G3.

## 3. Results and Discussion

### 3.1. Corrosion Behaviors of L360NB in Condensate and the Role of CO_2_

**Figure 1:**
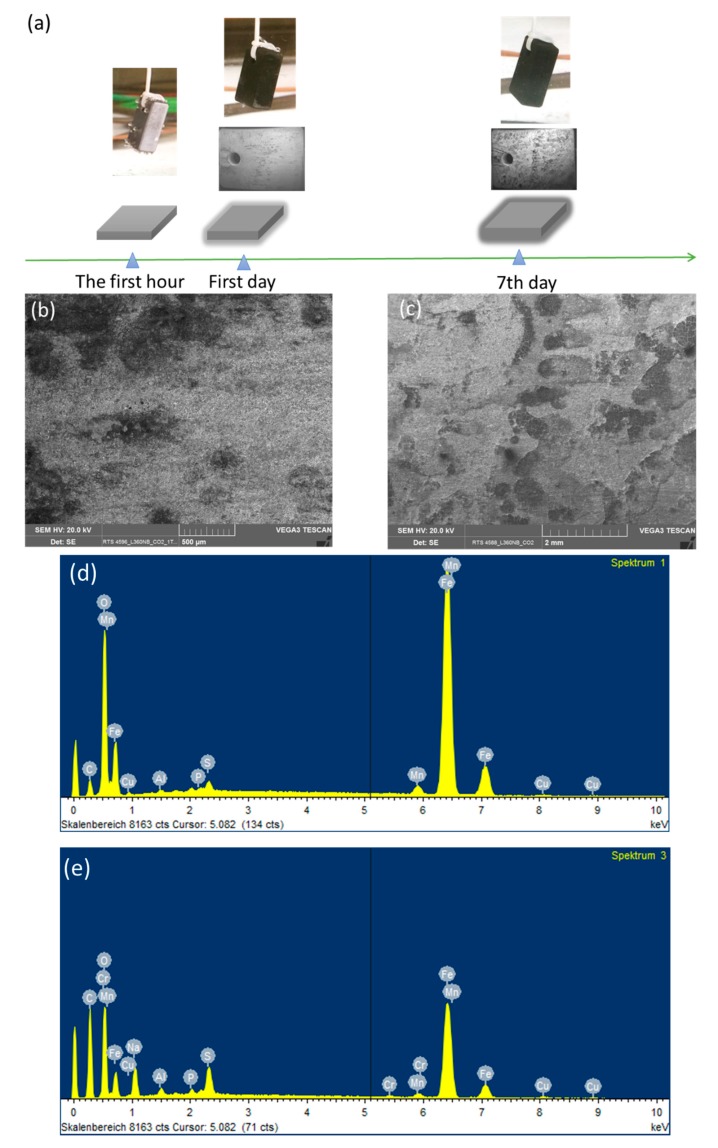
Corrosion behaviors of L360NB after 1 (**b**,**d**) day and (**c**,**e**) 7 days of exposure to CO_2_ saturated “original” condensate at 278 K. (**a**) Illustration of corrosion process with scanning electron microscopy (SEM) overview images of corroded coupons at the corresponding time. (**b**,**c**) are SEM images and (**d**,**e**) are energy-dispersive X-ray spectroscopy (EDS) spectra of corroded coupons. The condensate contained 0.114 M H_2_SO_4_ and 0.0184 M HNO_3_ (pH 2.13).

**Figure 2:**
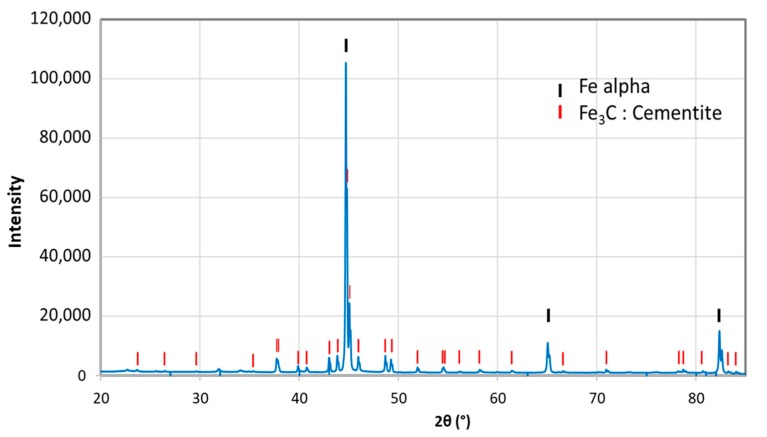
X-ray diffractometry (XRD) analysis of 7-day-exposed coupon in CO_2_ saturated condensate containing H_2_SO_4_ 0.114 M and HNO_3_ 0.0184 M.

So as to investigate the corrosion behaviors of pipeline steel (L360NB) in the CO_2_ saturated artificial condensate, freshly ground coupons were exposed and analyzed by SEM and EDS after 1 and 7 days. As shown in Figure 1, it was clear that the corrosion process occurred right from the beginning of exposure. EDS spectra further showed the chemical change in the corrosion product from 1 to 7 days of exposure. The atomic concentration ratio of the O and S peaks (more than 4 times) indicated the formation of FeSO_4_ (xH_2_O) on the surface as the main product. Besides those common peaks, the EDS of 1 day-exposed coupon interestingly showed no peak of Cr and a quite small peak of C, while the Cr peak appeared, and C peak became stronger in the case of 7-day-exposed coupon. The C peak is associated mainly with iron carbide Fe_3_C (cementite) left over due to the preferential dissolution of the α-ferrite phase within the steel microstructure. This was confirmed by XRD analysis (Figure 2).

The original composition of Cr in carbon steel L360NB is quite low, only 0.031 weight percent, thus the Cr peaks appeared in EDS, which accounts for more than 0.27 weight percent (calculated data from EDS), suggest the migration of Cr during corrosion process. This behavior is governed by the difference in diffusion rates of iron and chromium, and thus the components corroding at relatively slow rates will be enriched in the surface [17,18]. During the corrosion process, the main anodic reaction is the active dissolution of iron, and the other metal components, while the main cathodic reaction is the reduction of hydrogen ion, especially at starting pH 2.13. After a certain time, due to the difference between the diffusion rates, the outer region will be enriched in Fe, Mn and then Cr, whereas for the region closet to the metal/film interface, the composition corresponds to that of bulk metal. Thus, the result suggested is that the enrichment of Cr is kinetically, on the first day, insufficient for building up the significant content in the outer surface. The enrichment of Cr on the surface of corroded coupons in this case, however, is not related to a building up a passive layer but rather a clue or an indicator for kinetical information of corrosion process.

**Figure 3:**
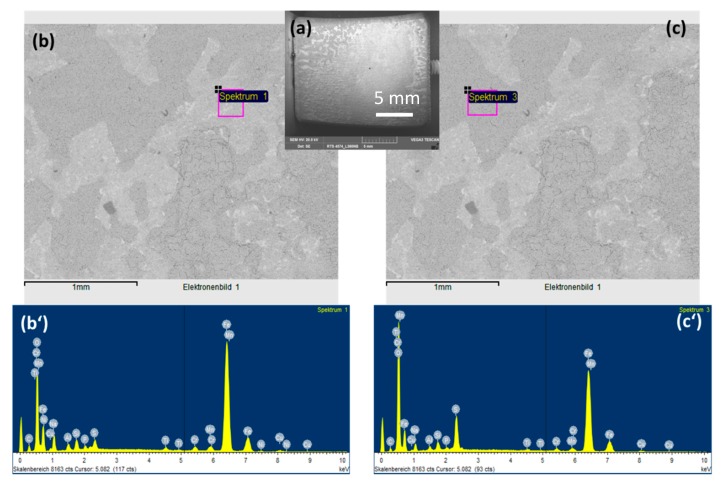
SEM images (**a**,**b**,**c**) and EDS spectra of brighter (**b’**) and darker (**c’**) areas of 7-day-exposed L360NB in Ar saturated condensate.

To observe the CO_2_ effects outside the strong acidic condensate on the corrosion behaviors of L360NB, the same experiment was done with the saturation of Ar, instead of CO_2_. SEM images clearly show less porous and thinner corroded surface and EDS data of the 7-day exposed coupon show Cr and C peaks, together with a strong peak of S, suggesting that the sulfate product was formed (Figure 3). C peak intensity was weaker than that from CO_2_ exposed coupon, indicating less iron dissolution and therefore, less left-over cementite. 

**Figure 4:**
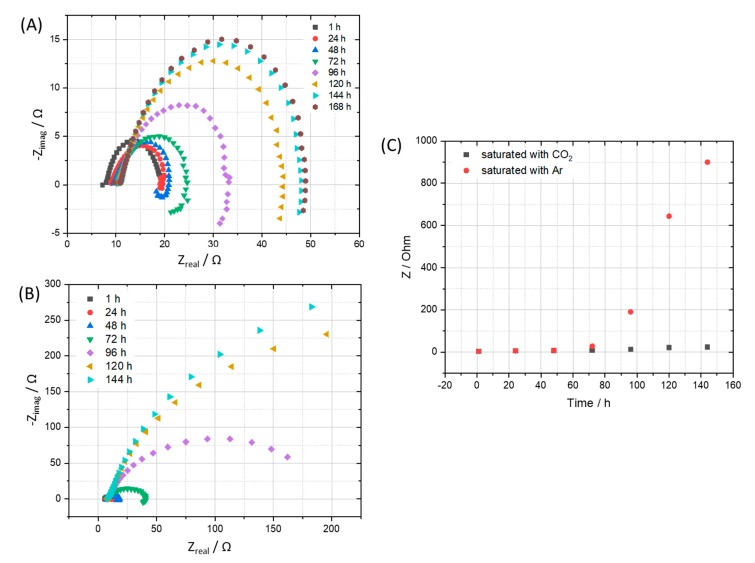
EIS spectra of coupon exposed to the condensate that saturated with (**A**) CO_2_ in comparison with (**B**) Ar and corresponding calculated corrosion resistance (**C**).

Electrochemical impedance spectra were recorded simultaneously during the exposure test, showed further evidence of the corrosive role of CO_2_ compared to that of Ar. The corrosion process along with the building up of the corrosion product film, resulting in an increase of corrosion resistance as can be seen clearly in the impedance spectra and the calculated corrosion resistance (Figure 4). After 7 days, the corrosion process happened on the coupon that exposed to Ar is slowing down indicated by a much higher corrosion resistance value, while the one exposed to the CO_2_ saturated condensate still actively corroded. A closer look at the corrosion resistance clearly shows that during the first 2 days, both the coupons exposed to Ar and CO_2_ saturated condensates got the same corrosion resistance, suggesting that the main cathodic reaction is the reduction of hydrogen ion that dissociated from HNO_3_ and H_2_SO_4_. After that, the corrosion resistance of the coupon exposed to Ar saturated condensate increased while the one exposed to CO_2_ continued to corrode actively. The result is suggesting that, after the protons from acids were consumed, the CO_2_ role in terms of cathodic reaction became dominant:

(1) $$H_{2}{\mathrm{CO}}_{3}\;+\;e^{-}\;\to \;\frac{1}{2}H_{2}\;+\;{\mathrm{HCO}}_{3}^{-}$$

Consequently, H_2_CO_3_ serves as an additional source of H^+^ ions that results in a higher rate of hydrogen evolution reaction than that of the Ar saturated condensate case. Furthermore, H_2_CO_3_ may adsorb and react on the electrode surface, resulting in the extended active time until a stable corrosion film can be established [19,20,21,22]. It is noteworthy to mention that different from previous studies, which were carried out at a higher temperature (>323 K), after a month of exposure test at 278 K, XRD analysis did not reveal any crystal FeCO_3_ phase, representing a stable and possible semi-protective film on the exposed steel coupon. The absence of FeCO_3_ in the corrosion products on carbon steel which resulted from liquid CO_2_ with impurities at 278 K and 5 MPa was also confirmed in a recent separated study [23]. It is therefore expected that there might be no protective layer formed in the presence of condensate at 278 K. 

In summary, when condensate is formed, corrosion process happens due to acidifying. The pH will then have increased since (1) the acids are consumed and (2) alkaline products have been produced. Furthermore, (3) there are no/low reactant(s) for the cathodic reaction. Due to (1), (2), and (3), the corrosion declines to low levels for Ar-experiment, while it may continue for CO_2_ experiment since CO_2_ is continuously supplied to the test system.

### 3.2. Effects of Condensate Concentration

**Figure 5:**
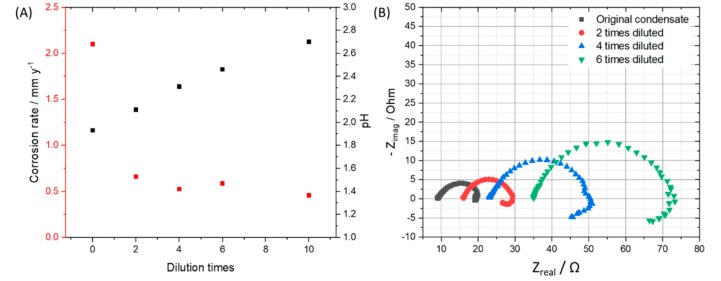
(**A**) Corrosion rates of the 7-day-exposing L360NB coupons in original, 2, 4, 6, and 10 times diluted condensates and pH of these CO_2_ saturated condensates at the beginning of the test. (**B**) EIS spectra of 24 h exposed coupons in original and diluted condensates. Original condensate contains 0.114 M H_2_SO_4_ and 0.0184 M HNO_3_.

**Figure 6:**
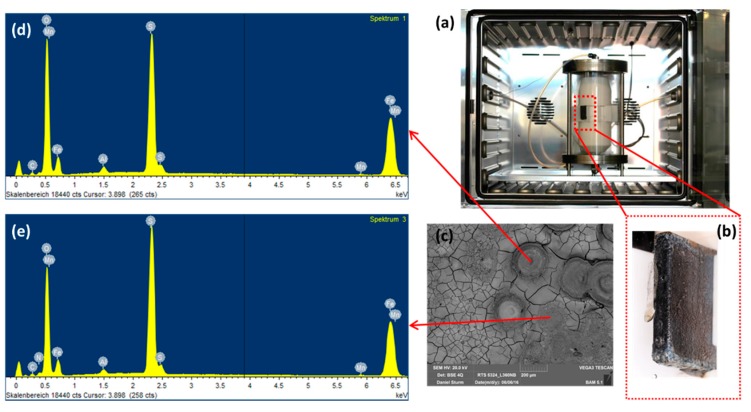
Gaseous reactor (**a**) with the L360NB coupon attached on the Teflon case (**b**) and the (**c**) SEM and (**d**,**e**) EDS spectra of the coupon after 7 days exposing to CO_2_ gas with 6 times reduced NO_2_, SO_2_, and O_2_ and same H_2_O concentration.

To observe the effects from the concentration of impurities without changing the ratio among components, we diluted the original condensate (0.114 M H_2_SO_4_ and 0.0184 M HNO_3_) 2, 4, 6, 10 times and performed exposure test, then calculated the mass loss of the coupons in each case. By diluting the original condensate 2, 4, 6, and 10 times, the pH of the solution after saturating with CO_2_ was changed from 1.93 to 2.11, 2.31, 2.46 and 2.7, respectively. After 7 days of exposing, under the CO_2_ saturation condition, all the solutions have the pH more than 5. From weight loss data and the calculated corrosion rate, it is clear that the 2-time-diluted condensate caused much slower corrosion process than that of the original one, but there is not so much difference among 2, 4, 6, 10-time-diluted condensate (Figure 5A). This result indicates that when the initial pH is less than 2, the corrosion rate is high, and when it is between 2 and 3, the corrosion rate is reduced about 4 times compared to that of the pH 2. To clarify the influence of pH on the corrosion rate and corrosion mechanism, SEM and EDS of all samples after 7 day-exposure tests were taken. Interestingly, although the corrosion rates are not so much difference, the EDS data of the coupons that exposed to the 6- and 10-times diluted condensates showed no peak of Cr, which is similar to the EDS of the coupon that exposed to the original condensate for one day. This result suggests that when the condensate concentration is 6 times diluted, the corrosion kinetic is significantly changed, Fe dissolution and therefore Cr enrichment is slower. To confirm this “6 times benchmark” data, the exposure test in the gaseous reactor was carried out with the CO_2_ gas stream containing 6 times reduced concentration of the impurities (166.7 ppm_v_ NO_2_, 36.7 ppm_v_ SO_2_, 0.3% O_2_) and the same temperature (278 K), flow rate (1.5 L min^−1^) and concentration of other components (2.5% H_2_O). The EDS data showed no peak of the Cr, which is in agreement with the result from the synthetic condensate exposed coupons (Figure 6).

### 3.3. Effects of Each Acidic Component in Condensate on the Corrosion Behavior

#### 3.3.1. Role of Each Component on the Surface Morphology and Corrosion Rate

In order to clarify the role of each acidic component in the condensate, and therefore the role of each impurity in CO_2_, two separate single component condensates, H_2_SO_4_ 0.114 M and HNO_3_ 0.0184 M, with the same concentration as in the original condensate were prepared. The pH was then adjusted to 2.13 so that the initial pH is the same as in the original one, and then the exposure tests were carried out, as well as corrosion potential, impedance, pH, conductivity measurements during 7 days. 

**Figure 7:**
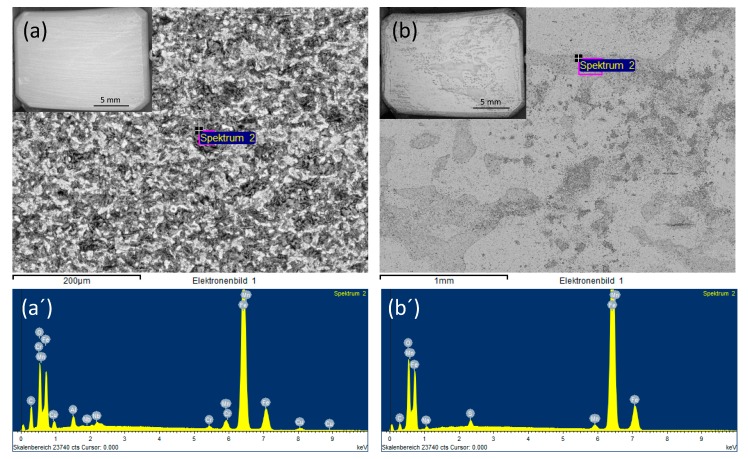
Backscatter electron (BSE) and EDS spectra of L360NB exposed to (**a**,**a’**) 0.0184 M HNO_3_ and (**b**,**b’**) 0.114 M H_2_SO_4_ contained condensates.

**Figure 8:**
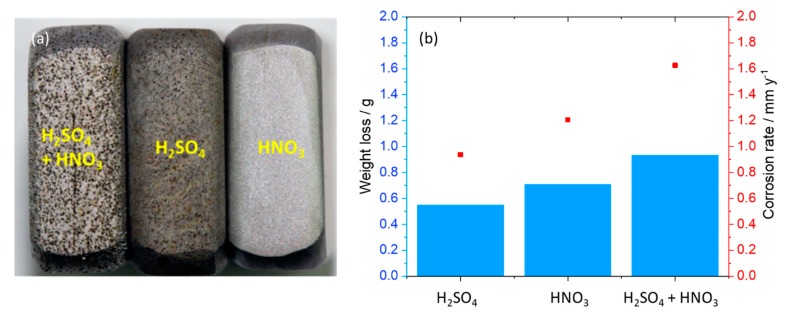
(**a**) Surface of corroded coupons (on the sides) exposed to the mixture, H_2_SO_4_ and HNO_3_ condensates, relatively (from left to right) after cyclic voltammetry tests and (**b**) corrosion rate calculated from weight loss after one-month exposure test. The initial weight of each coupon was around 13 g.

SEM images of the coupon exposed to 0.0184 M HNO_3_ condensate showed homogenous corrosion, while the one exposed to 0.114 M H_2_SO_4_ condensate got the pitting on the sides and localized corrosion on the main surface (Figure 7). This phenomenon is in agreement with the literature, that sulfates are present at or close to the film surface, therefore leading to pitting initiation and later on pitting corrosion [18]. Further one-month exposure tests, as well as electrochemical characterization including cyclic polarization (CV) and potentiodynamic, were carried out to verify this phenomenon. Figure 8 clearly shows that even under the applied potentials up to 500 mV in the case of CV, and 1 V in the case of potentiodynamic test, the surface of the coupon that exposed to HNO_3_ showed no pitting corrosion, while strong pitting corrosion was found in the case of H_2_SO_4_, and the mixture of both did cause broader and deeper pits with faster corrosion rate than that of the single component condensate. Furthermore, the same corrosion types were observed from a one-month exposure test (Figure 8). The corrosion rate calculated based on the weight loss again confirmed stronger corrosive effects of the mixture than that of HNO_3_ and of H_2_SO_4_. 

**Figure 9:**
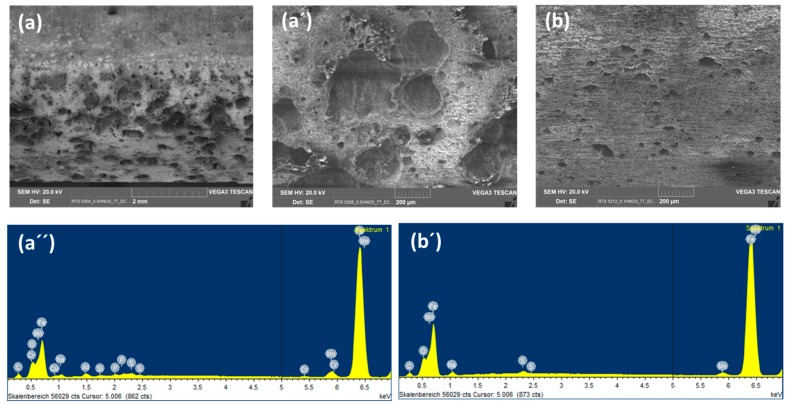
EDS and SEM images of L360NB coupon exposed to the condensates containing 0.114 M H_2_SO_4_ and (**a**,**a’**,**a”**) 2 times lower concentration of HNO_3_ (9.2 mM) and (**b**,**b’**) 10 times lower concentration of HNO_3_ (1.84 mM).

**Figure 10:**
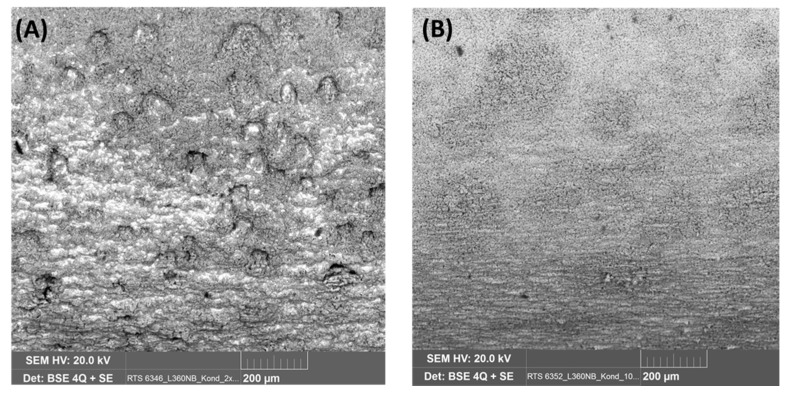
SEM images of L360NB exposed to the condensates containing 0.0184 M HNO_3_ and (**A**) 2 times lower H_2_SO_4_ concentration (57 mM), and (**B**) 10 times lower H_2_SO_4_ concentration (11.4 mM).

**Figure 11:**
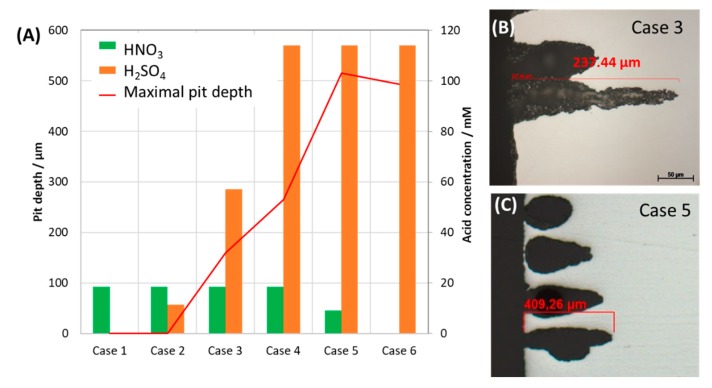
Effect of individual acid concentration on the maximal pit depth (**A**) of the exposed coupons and the cross-section images (**B**,**C**) of the 7-day exposed coupon, showing example of pit appearance. The condensates contained varied concentration of H_2_SO_4_ and HNO_3._

Further investigation on the role of HNO_3_ on the corrosion form was done with the same concentration of H_2_SO_4_ and 2 and 10 times lower concentration of HNO_3_ and vice versa. After 7 days of exposure, the coupons were analyzed by SEM, EDS and the pit depth were measured. The results were presented in Figure 9 and Figure 10. As can be seen from SEM images, although HNO_3_ alone caused no pitting corrosion, together with the same amount of H_2_SO_4_, more HNO_3_ will cause bigger but shallower pits and stronger localized corrosion (Figure 9a,a’). The pit was not deepest in the case of the original mixture, but with half of the HNO_3_ and same H_2_SO_4_ concentration as compared to that of the original mixture. On the other hand, when the concentration of HNO_3_ is kept the same as in the original condensate, and the H_2_SO_4_ is 10 times reduced, no pitting corrosion was observed. A summary of the maximal pit depth on the concentration of each acidic component is shown in Figure 11. 

The results suggested that the pitting behavior of carbon steel L360NB was strongly depended on the ratio of HNO_3_/H_2_SO_4_ and therefore the ratio of NO_2_/SO_2_ concentration in CO_2_ gas stream. These impurities are unavoidable; however, the pitting process could be mitigated by simultaneously decreasing their concentration and increasing the ratio of NO_2_/SO_2_. 

#### 3.3.2. Kinetic of Corrosion Process

**Figure 12:**
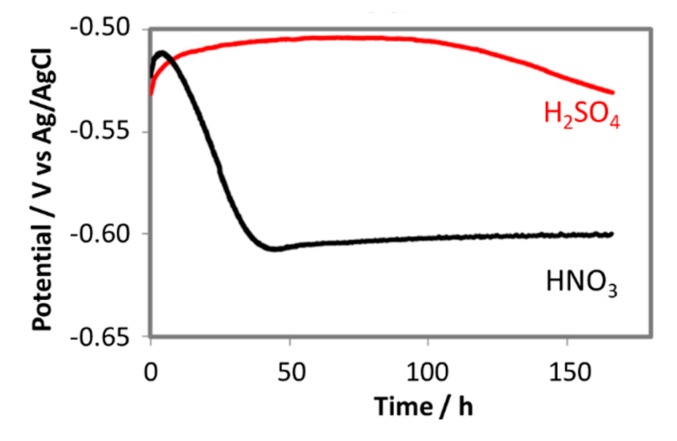
The change in free corrosion potentials of L360NB exposed to CO_2_ saturated 0.114 M H_2_SO_4_ and 0.0184 M HNO_3_ contained condensates.

**Figure 13:**
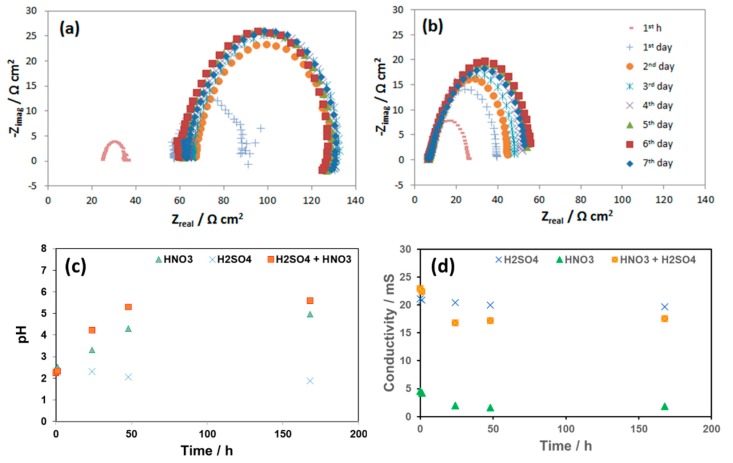
EIS spectra of L360NB during 7 days of exposing to CO_2_ saturated (**a**) 0.0184 HNO_3_ and (**b**) 0.114 M H_2_SO_4_ contained condensates and (**c**) the pH and (**d**) conductivities of these condensates during the reaction time in comparison with that of the original condensate.

The change in free corrosion potentials between the coupon exposed to 0.0184 M HNO_3_ and 0.114 M H_2_SO_4_ is a clear evidence of these acidic individual effects on the corrosion process of the pipeline steel (Figure 12). Although the initial pH in both cases was the same (2.13), the coupon exposed to HNO_3_ did show more negative corrosion potential than that of the coupon exposed to H_2_SO_4_, suggesting more active corrosion process. In the case of HNO_3_ contained condensate, the corrosion potential decreased significantly during the first 2 days, and then stabilize afterward, while in the case of the H_2_SO_4_ contained condensate, the OCP decreased much slower on the first 4 days and then faster after that time. EIS data taken during these 7 days further gave us more details on what happened on the surface of corroded coupons (Figure 13).

**Figure 14:**
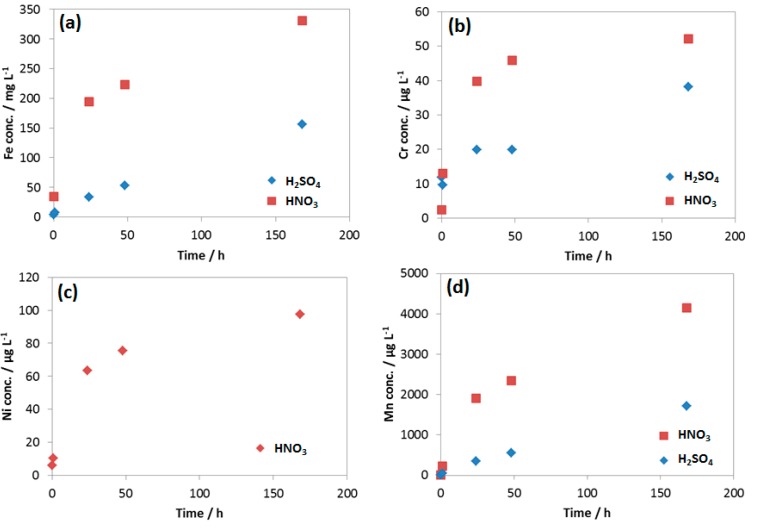
Concentration of (**a**) Fe; (**b**) Cr; (**c**) Ni and (**d**) Mn in the reacted 0.0184 M HNO_3_ and 0.114 M H_2_SO_4_ condensates.

As seen from the EIS spectra the changes in corrosion resistance happened significantly in the first hour, the first and the second days. The same phenomenon happens with the pH and the conductivity in the case of HNO_3_ contained condensate. After 2 days, the pH reached the value more than 4, while in the case of H_2_SO_4_, pH stayed around the initial value. This changing pattern of pH is in agreement with the OCP change discussed above. During these 7-day exposure tests, the reacted solutions were collected and analyzed by AAS (Figure 14). 

The results showed that, although the concentration of HNO_3_ is 6 times lower than that of H_2_SO_4_, the concentration of Fe, Mn and Cr from the HNO_3_ contained condensate was double than that of H_2_SO_4_ one, indicating much faster Fe, Mn and Cr dissolution rate caused by HNO_3_. This was in agreement with the result from weight loss test and the corresponding corrosion rate presented in Figure 8. It is interesting that Ni was not detectable in the case of the reacted solution that contained H_2_SO_4_, but it was detected in HNO_3_ contained condensate with the concentration higher than Cr, even though the original concentration in coupon was the same (0.03%). Thus, it is clearly shown that the initial proton concentration or pH did not entirely determine the rate of reaction; it is the nature of anion that strongly involved here. As mentioned, EDS data showed no peak of Cr in the case of the coupon exposed to H_2_SO_4_, while the peak of Cr in the case of the coupon exposed to HNO_3_ can be observed (Figure 6). This is in agreement with the pH data and the Fe, Cr, Ni, Mn dissolution rate. 

### 3.4. Effects of Flow Rate, Microstructure of Steel and Renewal of Condensate on Pitting Behavior

#### 3.4.1. Effects of CO_2_ Flow Rate and Microstructure of Steel

**Figure 15:**
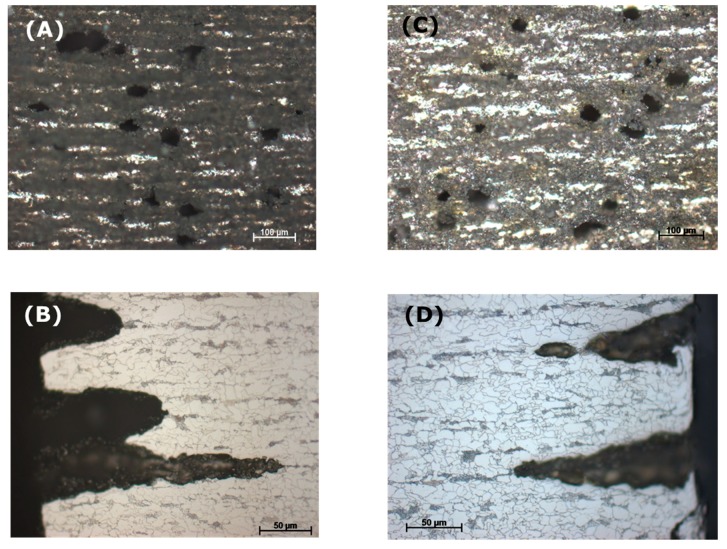
Effect of CO_2_ flow rate on the pitting behavior of L360NB in condensate containing 0.0184 M HNO_3_ and 0.114 M H_2_SO_4_. (**A**,**B**) 50–60 mL/min, and (**C**,**D**) 20–30 mL/min. (**B**) and (**D**)are the etched cross-section image of (**A**) and (**C**), respectively.

To observe the effect of flow rate on the corrosion process inside the steel structure, the CO_2_ flow was adjusted to be between 50–60 mL/min, in comparison with that at 20–30 mL/min in the same condensate. It was confirmed from the XRD analysis of the corrode coupon that the ferrite structure in the steel was reacted and dissolved, resulting in a cementite layer as black pulver on the coupon surface. The etched cross-section image of the corroded coupon shown in Figure 15 again proved this suggestion. For both cases, low or high flow rate, the acid selectively reacted with the ferrite, following the steel microstructure. This also explains why the coupon just got pitting corrosion on the side, not on the main surface. Figure 15 shows that the higher flow rate induced broader and deeper pits than that of the lower rate.

**Figure 16:**
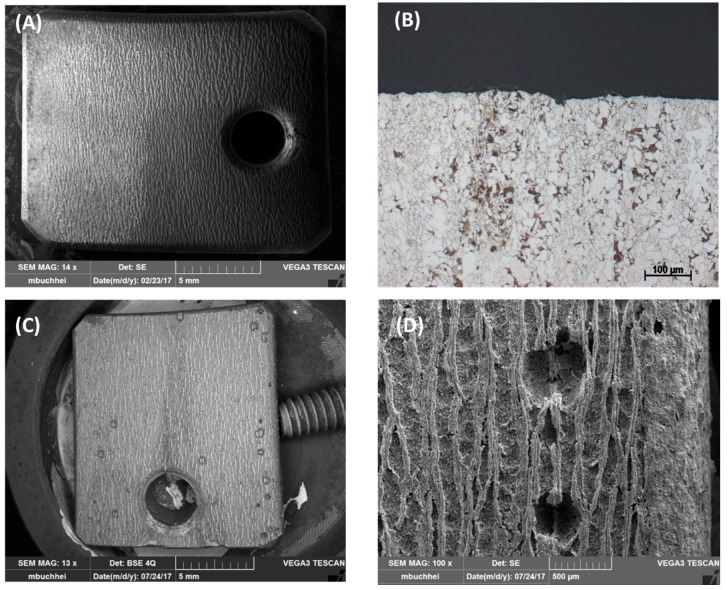
Pitting behavior of L485MB after exposing to (**A**,**B**) condensate containing 0.0184 M HNO_3_ and 0.114 M H_2_SO_4_ in comparison with L485MB exposed in (**C**,**D**) 0.114 M H_2_SO_4_ condensate. (**A,C,D**) are SEM images and (**B**) is cross-section image of etched (**A**) coupon. All the coupons were exposed for 7 days, under CO_2_ saturation condition and at 278 K.

To further confirm the dependency of pitting behavior on the steel microstructure, another commercial pipeline steel, carbon steel L485MB, was chosen to compare with L360NB since it has the same element content as L360NB, but different in microstructure [24]. As observed in Figure 16A,B, owing to its microstructure that has less banding structure, pitting happened at less intensity. However, stronger ferrite dissolution was observed, leaving behind thicker layer of cementite as compared to that of L360NB. After 7 days exposing to the 0.114 M H_2_SO_4_ condensate, L485MB suffered from strong pitting corrosion on the surface (Figure 16C) with the pit depth around 100 µm but much broader (up to 500 µm wide) as compared to that in case 5 (Figure 11) of L360NB coupon tested in the same condition. 

#### 3.4.2. Effects of Condensate Renewal

**Figure 17:**
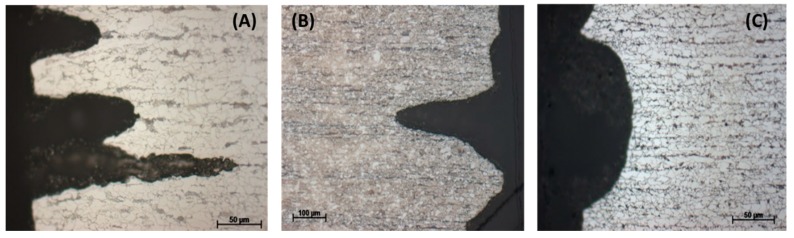
Effects of renewal condensate during 7 days of exposure tests. (**A**) Without changing the condensate, (**B**) with the change of condensate on the 3rd day and (**C**) with the changes of condensate on 2nd and 5th days. Condensate contains 0.0184 M HNO_3_ and 0.114 M H_2_SO_4_.

To observe the effect of renewal condensate on the pitting behavior of carbon steel, seven-day exposure tests were carried out without and with one time changing, 2 times changing of the condensate. The new condensate was pre-cooled and saturated with CO_2_, then was pumped into the testing flash under CO_2_ atmosphere. Figure 17 presents the cross-section images of the exposed coupon after etching. Without changing the condensate, the pit width is smaller than that of one and two times changing. The pit depth, however, is deeper in case of non-changed condensate and the pit formed following the corn structure. With the renewal of condensate, it can be expected that the newly provided proton reacts strongly with the active corroded surface not only inside the pits but also all over the coupon surface, resulted in bigger and broader pits. This effect was significant in the case of two times changing in comparison with only one time changing the condensate. The increased pitting process due to condensate renewal further suggested that the corrosion products, as well as the left-over cementite layer, have no protectability against newly formed acidic condensate. 

## 4. Conclusions

The roles of SO_2_ and NO_2_ in CO_2_ stream in the corrosion mechanism of carbon steel L360NB were investigated by electrochemical characterization and exposure tests at 278 K using CO_2_ saturated synthetic condensate made of HNO_3_ and H_2_SO_4_. It was shown that when the condensation of SO_2_ and NO_2_ with water in the form of H_2_SO_4_ and HNO_3_ happens, leading to the pH less than 2, the corrosion rate can be more than 2 mm per year. In the presence of condensate, hydrogen evolution reaction happens with the proton provided by the condensate, until the pH raises to value of 4, the proton will be further supplied by CO_2_. Further results from the varying concentration of acidic component while keeping the pH value at 2.13, however, did show the different corrosion mechanism and processes indicating a strong influence of each anion in the synthetic condensate. Both electrochemical and exposure tests showed strong pitting and localized corrosion happened in H_2_SO_4_ contained condensate, while HNO_3_ induced only general corrosion. The pitting behavior was shown to be governed by the ratio of HNO_3_/H_2_SO_4_, the microstructure of the exposed metal, gas flow rate, and the renewal of condensate. In all cases, at 278 K, under the continuous presence of condensate, no FeCO_3_ could be formed, and the corrosion products resulted from condensate are not protective to the pipeline steel, strongly indicate the need for continuous control of gas quality, as well as a careful choice and design of transport pipeline construction materials. 

## Figures and Tables

**Table 1:** Chemical composition with major elements ^1^ as weight percent of the investigated material.

C	Si	Mn	Cr	Cu	Ni	Al	P	S	Mo	N
0.126	0.094	1.303	0.031	0.029	0.029	0.036	0.0142	0.006	0.003	0.008

^1^ Fe as balance.

**Table 2:** Experimental condition.

Materials	Carbon Steel L360NB (comparable to X52) and L485MB (X70)
Purging gas and flow rate	Pure CO_2_ (compared to pure Ar), 50–60 mL/min
Testing temperature, pressure	278 K, <1 bar
Aqueous phase	500 mL synthetic condensate made of varied concentration of H_2_SO_4_ and HNO_3_ pH was adjusted by adding NaOH to 2.13. Prior to immersing of metal coupons, the solution was purged with Ar for 30 min and then saturated with CO_2_ (pH ≈ 1.9).
